# HIV-1 recombinants with multiple parental strains in low-prevalence, remote regions of Cameroon: Evolutionary relics?

**DOI:** 10.1186/1742-4690-7-39

**Published:** 2010-04-28

**Authors:** Jean K Carr, Nathan D Wolfe, Judith N Torimiro, Ubald Tamoufe, E Mpoudi-Ngole, Lindsay Eyzaguirre, Deborah L Birx, Francine E McCutchan, Donald S Burke

**Affiliations:** 1 Institute of Human Virology, Univ. of Maryland School of Medicine, Baltimore, MD, USA; 2 Global Viral Forecasting Initiative, San Francisco, and Stanford University, Program in Human Biology, Stanford, CA, USA; 3 Faculty of Medicine and Biomedical Sciences, University of Yaounde I, Yaounde, Cameroon; 4 Chantal Biya International Reference Centre, Yaounde, Cameroon; 5 Hopital Militaire de Yaoundé, Yaounde, Cameroon; 6 Global AIDS Program, CDC, Atlanta, GA, USA; 7 Bill and Melinda Gates Foundation, Seattle, WA, USA; 8 University of Pittsburgh Graduate School of Public Health, Pittsburgh, PA, USA

## Abstract

**Background:**

The HIV pandemic disseminated globally from Central West Africa, beginning in the second half of the twentieth century. To elucidate the virologic origins of the pandemic, a cross-sectional study was conducted of the genetic diversity of HIV-1 strains in villagers in 14 remote locations in Cameroon and in hospitalized and STI patients. DNA extracted from PBMC was PCR amplified from HIV(+) subjects. Partial *pol *amplicons (N = 164) and nearly full virus genomes (N = 78) were sequenced. Among the 3956 rural villagers studied, the prevalence of HIV infection was 4.9%; among the hospitalized and clinic patients, it was 8.6%.

**Results:**

Virus genotypes fell into two distinctive groups. A majority of the genotyped strains (109/164) were the circulating recombinant form (CRF) known to be endemic in West Africa and Central West Africa, CRF02_AG. The second most common genetic form (9/164) was the recently described CRF22_01A1, and the rest were a collection of 4 different subtypes (A2, D, F2, G) and 6 different CRFs (-01, -11, -13, -18, -25, -37). Remarkably, 10.4% of HIV-1 genomes detected (17/164) were heretofore undescribed unique recombinant forms (URF) present in only a single person. Nearly full genome sequencing was completed for 78 of the viruses of interest. HIV genetic diversity was commonplace in rural villages: 12 villages each had at least one newly detected URF, and 9 villages had two or more.

**Conclusions:**

These results show that while CRF02_AG dominated the HIV strains in the rural villages, the remainder of the viruses had tremendous genetic diversity. Between the trans-species transmission of SIV_cpz _and the dispersal of pandemic HIV-1, there was a time when we hypothesize that nascent HIV-1 was spreading, but only to a limited extent, recombining with other local HIV-1, creating a large variety of recombinants. When one of those recombinants began to spread widely (i.e. became epidemic), it was recognized as a subtype. We hypothesize that the viruses in these remote Cameroon villages may represent that pre-epidemic stage of viral evolution.

## Background

The geographic location of the origin of the HIV-1 pandemic is Central West Africa, where cross-species transmission of SIV_cpz _occurred from chimpanzee (*Pan troglodytes troglodytes*) to human [1-3]. From that transmission event the virus adapted into group M HIV-1 and gradually spread throughout the world. The genetic forms of HIV-1 currently present in Central West Africa, ground zero of the pandemic, may shed light on those early events.

Characterization of HIV-1 genetic diversity in different regions of the world is a challenging, on-going effort. Phylogenetic analyses of viral sequences have revealed distinct monophyletic clusters of strains called subtypes. There are now 9 official subtypes, and over 45 validated circulating recombinant forms (CRF) http://www.hiv.lanl.gov/, and they exist in different patterns in various regions of the world. These patterns have been moderately well described, with strains from most countries now characterized to some degree or another. Cameroon is a location where the genetic diversity has been repeatedly studied; there have been at least 8 scientific reports of the genetic subtypes in Cameroon since 2005 [4-12]. The reason for this intense interest is that the HIV epidemic in Cameroon presents a paradoxical picture: the prevalence of infection is not high by African standards (<10% in rural areas), yet the genetic diversity, including multiple recombinants of complex structure, is extremely high. While all of these studies have reported CRF02_AG as the most prevalent single genetic form in circulation, with estimates ranging from 45% to 61%, the remaining strains have consisted of an array of other genetic forms, both classifiable and not [4-13]. Because of this genetic diversity, partial genome sequencing of relatively small sample sets has hampered the description of the epidemic fully. This report presents the genetic subtypes of more strains from rural Cameroon than have previously been reported, using nearly full genome sequencing to more completely describe the many unique recombinant forms (URF).

## Results

### Partial Pol Analysis

**Figure 1:**
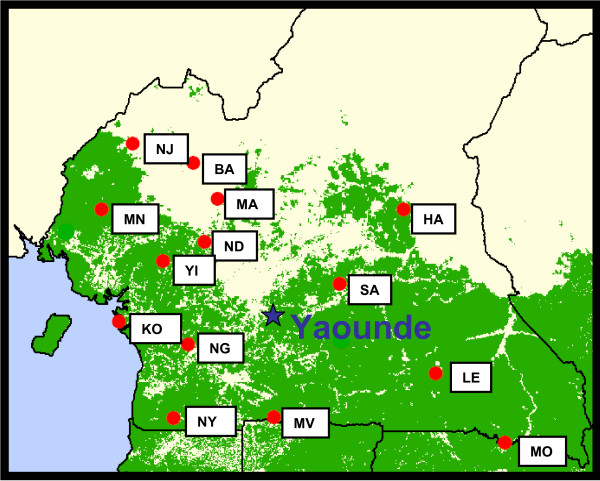
**Satellite Map of Cameroon with study sites**. Study sites are indicated by the two letter code identifying them.

There were 164 HIV-1 strains characterized by partial *pol *sequencing out of a possible 178. Most of the strains {126 (76.8%)} were collected from 14 rural villages, where the prevalence of infection ranged from 1.9% to 7.5%, though there was one site with a prevalence of 16.3%. The geographic location of the study sites, identified by 2-letter code, is shown in Figure 1. In addition to samples from the rural villages, 26 strains from STI out-patients or general medicine in-patients were characterized, among whom the prevalence of infection was 8.6%. Finally, 12 strains were examined from discarded blood units from the blood bank in Yaounde, the capital of Cameroon. No non-group M strains were found.

**Figure 2:**
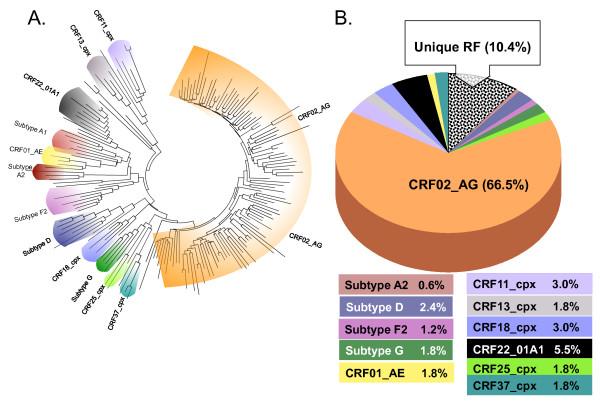
**Panel A: Phylogenetic analysis of 164 partial *pol *sequences from Cameroon**. A neighbor-joining analysis of partial *pol *sequences (protease and the amino terminus of RT) using the Kimura 2-parameter method of distance calculation was performed with representative strains of varying subtypes and CRF (identified by name). The scale bar indicates genetic distance of 1%. Nodes with significant bootstrap support (>70%) and containing Cameroon sequences are indicated with color. **Panel B**: **Relative proportions of different subtypes or CRF**. RF = recombinant form.

Phylogenetic analysis of the partial *pol *sequences showed that the majority (66.5%) clustered with CRF02_AG (Figure 2, panels A/B). Bootstrap analysis of the clusters was performed after the exclusion of URF, and significant bootstrap support (>70%) was present for the strains highlighted in the figure. The next most common genetic form, present in 5.5% of the strains, was CRF22_01A1. This CRF is a recombinant between sub-subtype A1 and CRF01_AE. Like CRF01_AE, CRF22_A101 is sub-subtype A1 in the pol region of the genome, but each of the CRFs (-01 and -22) form separate clusters within sub-subtype A1 that are distinct from each other, reflecting the different A1 strains that were parental to the different CRFs (Figure 2A). In addition, there were 10 more genetic forms identified: subtypes D and G, sub-subtypes A2 and F2 plus CRFs -01, -11, -13, -18, -25 and -37. About ten percent of the strains, however, could only be described as a wide variety of unique recombinant forms (URF). On the phylogenetic tree, they are scattered throughout the tree, reflecting their varied structure (Figure 2A). In addition to the subtypes and CRF listed already, these recombinants included those having regions from subtypes C, H and CRF09_cpx as well as regions that were unclassifiable.

**Figure 3:**
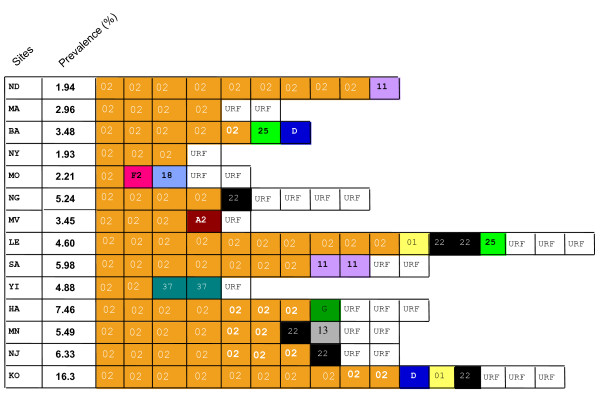
**Schematic showing the genetic types of viruses, based on partial *pol *sequences, by geographic site**. Each virus type is represented as a box, with the abbreviated name of the genetic form in the box. The two letter code for the site and the prevalence at the site are on the left. Letters = subtypes; numbers = CRFs.

The prevalence of the various genetic forms in the different rural sites is displayed in Figure 3, along with the overall prevalence at the site. The prevalence in the rural villages ranged from 2% to 16%, and the proportion of the strains that were URF ranged from 0% to 44%. Two sites (ND and BA) had no URFs, while others had 3 or more different ones (NG, LE, NJ and KO). There was no correlation, positive or negative, between the prevalence of infection and the proportion that were URF.

### Nearly Full Genome Analysis

**Figure 4:**
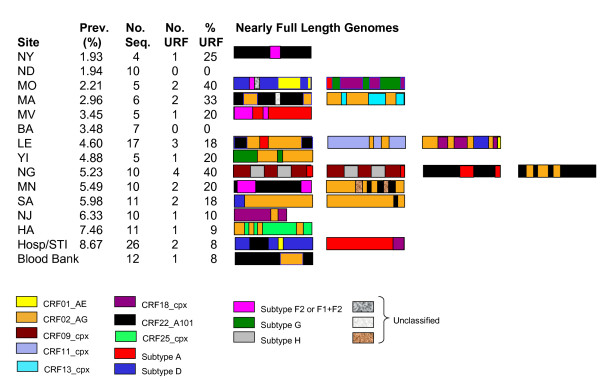
**Genetic structure of nearly full length unique recombinant genomes by site**.

Full genome analysis of a subset of the CRF02_AG strains and most of the non-CRF02_AG strains was performed. There were 78 full genomes completed in all, and of these 23 were URF. The diversity of forms is tremendous, even when the prevalence of infection is low (Figure 4). Diagrams of the subtype structure of the 23 URF that were sequenced in full for this study show that more than 10 different subtypes or CRF are included in the strains, plus regions that are impossible to classify (Figure 4). Presumably some of those regions are either new subtypes or CRF that have not yet been identified. One of the most striking of the sites is Lomie, where the prevalence is 4.6% but there were 3 different unrelated URF containing 7 different parental strains between the 3 of them; even a passing glance can detect the lack of relatedness between the different URF from that site. At only one site (NG) were there two complex URFs with the same structure, suggesting transmission linkage. Demographic information suggested that they were married, over 50 years of age, and self-described as monogamous.

Significant hypermutation was observed in 14.1% of the full genomes, and another 16.7% were otherwise defective. Most of the hypermutated strains were classified using Hypermut http://www.hiv.lanl.gov/[14], but a few were only partially hypermutated and were discovered by the characteristic G-to-A mutations. From the context of the hypermutations, APOBEC3G is the likely enzyme responsible. In one case, a hypermutated strain also had a 36 aa deletion in the *vif *gene, suggesting that, for that individual, APOBEC3G was unimpeded by *vif*. A little over half of the hypermutated strains were URF (6/11, 54.5%), while the rest were both CRF02_AG and the other genetic forms; the URFs, therefore, had a higher rate of hypermutation than the subtypes or CRFs. In addition to 11 hypermutated strains, there were 13 strains with major defects likely to make them functionally dead. Most were frame shift mutations leading to stop codons, but there were 3 strains that had large insertions/duplications in the *nef *gene. The sizes of the insertions or duplications were 17 aa, 40 aa and 75 aa, respectively. Defective genomes were not more prevalent in URFs than other genetic forms.

## Discussion

While genetic diversity in Cameroon has been described frequently in the literature, this report documents the high degree of genetic diversity using nearly full genome sequencing of samples from very rural sites in Cameroon. The prevalence of infection was relatively low by African standards (4.9%), and CRF02_AG was the predominant strain (66.5%), but about a third of the remaining HIV-1 genotypes detected (57/164) were confined to only one or at most a few persons. As others have shown, Central West Africa is the most likely geographical location for the origin for the HIV-1 pandemic [1-3]. However, counter-intuitively, the prevalence of infection there is lower than the newer epidemics to the east, west and south [15]. We hypothesize that the low prevalence is a reflection of lower transmissibility of HIV in these populations. Even in villages with a large number of unique recombinant forms (URF) such as LE, recombinants not only varied in structure but also in the parental strains involved. Only in one village were there 2 URFs with identical structures, probably sex linked; and the two major parental strains for that recombinant (CRF09_cpx, subtype H) were not even found in the population. Viral loads for these samples revealed no correlation between genetic form (CRF02_AG vs URF, for example) and level of circulating virus (data not shown), but there was a distinct lack of genetically related transmission pairs among the URF from the same village.

A low rate of transmissibility may explain the low prevalence, but it is very difficult to then account for the presence of recombinants with 4 or 5 different subtypes in one strain. Inter-subtype recombinants are generally the result of super-infection with different subtypes and are most commonly seen in populations with heavy exposure to multiple subtypes [16,17]. There are multiple subtypes in this population, but at a fairly low level. The hospitalized patients and STD clinic attendees would be expected to have the most risk, and the prevalence of infection among them was significantly elevated compared to the rural villages, but the rate of URFs among them was even lower than in subjects from the rural sites. Recent research among subjects in the capital, Yaounde, found that 16% of the subjects were dually infected with either 2 different subtypes or different strains from one subtype [9]. As with the subjects in this study, they were low risk. Further study in these populations is needed in order to discover what the mechanism behind this observation might be.

**Figure 5:**
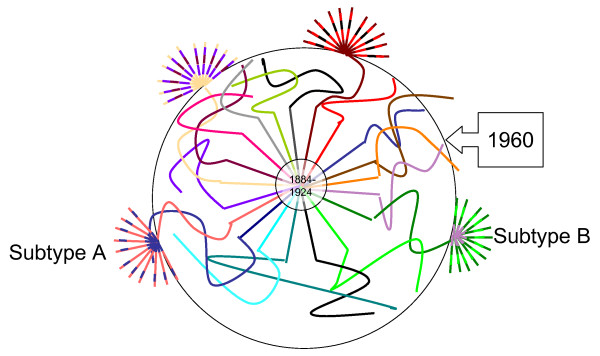
**Diagram of a hypothesized scenario of HIV emergence**. Different colors represent different strains of the virus. Distance from the center of the circle represents time since the initial emergence. Adapted from Kalish et al., 2004 [18].

A theoretical model to display one hypothesis is shown in Figure 5, an adaptation from Kalish et al., 2004 [18]. What is known about the origins of HIV put the date of transmission from chimpanzee to human between 1884-1924, the center of the figure [19]. We hypothesize that the original transmission was probably suboptimal in terms of replication in the human host and that the virus recombined extensively in its 'search' for the right combination of genetic factors. In the figure, this period of time is represented by multiple wavy lines. By 1960 in Kinshasa, there were two identifiable subtypes, or variants capable of epidemic spread, subtype A and subtype B/D [19,20]. They were designated as 'pure' subtypes because they were epidemically successful, even though they undoubtedly emerged from a long process of repeated recombination. In most countries in the world, subtypes have been introduced, and in some cases these have recombined to make circulating recombinant forms that are clearly descendants of those subtypes. The situation in Central West Africa is dramatically different from that picture, and we hypothesize that the reason for this is that the viruses still in circulation in rural areas of Cameroon resemble the pre-epidemic viruses, and that they are, in a sense, evolutionary relics.

## Conclusions

The genetic complexity of HIV strains from rural Cameroon defies both logic and experience. Multiply recombinant viruses are found in subjects who have a high risk of superinfection with different strains of HIV, such as commercial sex workers or injecting drug users. The village populations in this study, on the contrary, have a low risk of infection as captured by the prevalence, but harbor viruses having 3 or 4 different parental strains. Among the 78 viruses sequenced in full, there were at least 13 different subtypes, sub-subtypes or CRF represented. It is hypothesized that that this diversity may be due to remnants of the viruses predating the epidemic in 1960.

## Methods

### Subjects

Of the 17 village sites in Cameroon that were selected for this study, 14 were used for the genetic analysis, shown in Figure 1. In a study approved by the IRB of Johns Hopkins University, participants were healthy adults who gave consent to participate, most of them subsistence farmers and hunters [21]. In addition, in-patients at two district hospitals (Ndikinimeki, Lomie) and outpatients at STI clinics in those same locations were enrolled in the study. Finally, HIV-positive blood was collected from the central blood bank in Yaounde, Cameroon, to monitor genotypes among blood donors. Blood was drawn and plasma and peripheral blood mononuclear cells (PBMC) were separated using CPT blood collection tubes (BD, Inc, Franklin City, NJ). The plasma was tested for HIV antibodies by Ortho HIV-1/HIV-2 Antibody capture ELISA (Ortho-Clinical Diagnostics, Rochester, NY) and reactive samples were confirmed by two Western Blots (HIV Blot 2.2, Genelabs Diagnostics, Singapore and Calypte, Cambridge Biotech, Cambridge, MA). Those confirmed positive on both were used for viral load determination and DNA extraction. Viral load was measured using the Roche Amplicor HIV-1 monitor test, v. 1.5 (Roche Molecular Systems, Branchburg, NJ). High molecular weight DNA was extracted from the PBMC using QIAmp DNA extraction kits (Qiagen, Valencia, CA).

### PCR Amplification

The DNA from PBMC was amplified by nested PCR in the *pol *gene producing a 1.1 kb fragment spanning protease and part of the reverse transcriptase (RT) gene [22]. The first and second round reactions were conducted using 8 ml dNTP (1.25 mM), 5 ml 10× buffer (no MgCl_2_), 4 ml MgCl_2 _(25 mM), 0.5 ml of each primer (20 mM), 0.5 ml Ampli Taq gold (Applied Biosystems, Foster City, CA), water and template in 50 ml total reaction volume. First round cycling conditions were: 95°C, 10 min, then 45 cycles of 94°C 30 seconds, 55°C 30 seconds, 72°C 1.5 minutes, then 72°C 7 minutes. The first round primers were: Pro5F (5'-AGAAATTGCAGGGCCCCTAGGAA) and RT3474R (5'-GAATCTCTCTGTTTTCTGCCAG). The second round reaction was with Pro3F (5'-AGANCAGAGCCAACAGCCCCACCA and ProRT (5'-TTTCCCCACTAACTTCTGTATGTCATTGACA). The cycling conditions were the same except that the annealing temperature was 58°C and there were only 30 cycles.

Virtually full-length genomes of HIV-1 were amplified from selected strains based on the results of partial *pol *sequencing. Limiting template dilution into the first round was performed to decrease the complexity of the sample and allow for direct sequencing of the second round PCR product. The virtually full-length genome was amplified using MSF12b (5'-AAATCTCTAGCAGTGGCGCCCGAACAG) and OFMR1 (5'-TGAGGGATCTCTAGTTACCAGAGTC), followed by F2nst (5'-GCGGAGGCTAGAAGGAGAGAGATGG) and ofm19 (5'-GCACTCAAGGCAAGCTTTATTGAGGCTTA). PCR was performed as described [23,24], using the Expand Long Template kit (Boehringer-Mannheim) and a hot-start method with a melting wax barrier (Dynawax). Cycling conditions were: 94° for 2 min, then 10 cycles of 94°C for 10 s, 60°C for 30 s and 68°C for 8 min. This was followed by 20 cycles where the annealing temperature was 55°C. The final extension step was 68°C for 10 min. Multiple second round PCR amplifications were combined to provide sufficient template for sequencing.

### DNA sequencing

Template DNA for automated sequencing was prepared as described previously [23]. PCR amplification products of the *pol *gene and the nearly full-length strains were fully sequenced on both strands by using fluorescent dye terminators and an Applied BioSystems (Applied Biosystems Inc., Foster City, CA) Model 3100 DNA sequencer. DNA sequences were assembled using Sequencher software (Genecodes Inc., Ann Arbor MI) on Macintosh computers. All sequences had at least 2 clear readings in each direction for completion.

### Analysis

A multiple alignment of the Cameroon sequences with selected HIV-1 reference sequences was constructed using MacGDE 1.9.5, software based on Genetic Data Environment (GDE) adapted for Mac OS X [25,26]. Gaps that were introduced to create the alignment were eliminated in the final analysis. Reference isolates from the different subtypes and circulating recombinant forms from the pandemic, described in the National HIV Database at Los Alamos, NM, were used to classify the Cameroon sequences http://www.hiv.lanl.gov/. Phylogenetic trees were constructed using the neighbor-joining method and the consistency of branching order was evaluated using bootstrap analysis by MEGA3 software [27]. Genetic relationships can be obscured by the presence of recombinant or novel forms in the analysis of HIV-1 strains. To address this, phylogenetic trees were constructed that included only a few aberrant viral sequences at a time. Hypermutated sequences were identified using Hypermut 2.0 software from the National HIV Database http://www.hiv.lanl.gov/ and were deleted from appropriate analyses [14].

Recombinant analysis was done with bootscanning [28] and distance scanning [23] using SimPlot software, version 3.5[29]. The nucleotide positions of recombinant breakpoints were designated relative to HXB-2 (GenBank Accession No: K03455). The significance of the breakpoint assignment was assessed by the bootstrap value of the relevant node in the phylogenetic tree, which was >70% for significance.

### Nucleotide Sequences

Nucleotide sequence accession numbers of the *pol *gene sequences from Cameroon are available under GenBank Accession No. AY847362-AY847453. The nearly full length genomic sequences are available under GenBank Accession numbers AY371121-AY371170, GQ229529 and GU201494-GU201517.

## Competing interests

The authors declare that they have no competing interests.

## Authors' contributions

JKC supervised analysis and wrote paper. NW developed the study design, supervised sample collection and contributed to the analysis. JT, UT and EM supervised field teams conducting the study in the field and created the database. LE conducted genetic sequencing. DB and FM contributed to study design, laboratory oversight and analysis and DB conceived of the project and contributed at every stage.
